# Cæsarean Section

**Published:** 1861-01

**Authors:** 


*Cæsarean Section.*—Prof. B. F. Barker recently performed the Cæsarean operation at Bellevue Hospital, on account of a contracted pelvis, the antero-posterior diameter of the superior strait being only two inches, the cavity of the sacrum filled with a bony tumor. The child weighed nine pounds, and is still living. The mother died on the fifth day after the operation. (*American Medical Monthly*, *December.*) The Cæsarean section has also been performed twice within the last year, by Mr. Lorthioir, of Lalaing, France; in each case with success. The mothers are living and well. The result to the children is not stated.—(*Championniere's Journal.*)

